# Sensor Systems for FRP Lightweight Structures: Automotive Features Based on Serial Sensor Products

**DOI:** 10.3390/s19143088

**Published:** 2019-07-12

**Authors:** Linda Klein

**Affiliations:** Robert Bosch GmbH, Powertrain Solutions, Robert-Bosch-Straße 2, 71701 Schwieberdingen, Germany; linda.klein@de.bosch.com; Tel.: +49-711-811-17885

**Keywords:** sensor integration, signal analysis, condition monitoring, functionalized structures, fiber-reinforced polymer structures, automotive lightweight design

## Abstract

To achieve resource efficiency and an increased performance, as well as a higher safety and more features for vehicles, lightweight composites are a central sphere of activity for automotive innovations. This becomes particularly striking if the focus is not only a reduced vehicle weight but also an efficient overall concept. In addition to compatible material technologies and component design, new electronic solutions are of interest. A research contribution at the Robert Bosch Company deals with the direct integration of a current automotive acceleration sensor in fiber-reinforced polymer (FRP) parts. The sensor is part of the passive vehicle safety. Primarily, the principal application of the currently mounted sensor as an integrated part of the vehicle structure was proven. Sensor-integrated parts were evaluated on their sensing functionality as well as their structural performance. The present research is done to use the integrated sensor for a secondary feature. The study shows that the sensor can also be an indicator for the condition of its surrounding FRP structure. Hence, the sensor integration makes it possible to derive a secondary feature for automobiles by using the current sensor for future functionalized lightweight structures.

## 1. Introduction

To achieve resource efficiency and an increased vehicle performance, the automotive construction increasingly deals with lightweight design. For optimal solutions by compatible material technologies and a systematic component design, new approaches in the commodity field are using fiber-reinforced polymers (FRP). The potential of FRP structures lies not only in the possible weight reduction of the load bearing structure, but also in the set-up in layers of the composite can be used to integrate functional components, as for example electronics, directly into a part. The sum of the single components plus the vehicle body to which they are applied are often much heavier than the integrated structure; the integration allows secondary weight saving. A great benefit of the approach is the possibility to integrate sensors into an FRP automotive part. The number of automotive sensors increases due to increasing vehicle features, while the available construction space of the vehicle is restricted. This makes the approach favorable for future automotive construction.

## 2. Structure Integrated Sensors

A number of approaches exist for the integration of sensors in FRP structures. Applications of sensor-integrated parts are mainly in the areas of aviation, astronautics, mechanical engineering, robotics, wind energy and offshore applications. The integrated sensors fulfill functions such as process monitoring during the production of the structural parts, as well as the measuring of loads or the flow of air affecting the structure during operation. Another important function during operation is structural health monitoring (SHM). Since FRP lightweight design has already been established for aircraft construction, the development of sensors particularly for an integration in FRP structures has been promoted. The following section gives a short overview on decisive sensor technologies, especially for load measurement and damage detections.

### 2.1. Current State of Research

For the sensor technologies which are integrated in FRP structures, piezoceramic transducers (PZT) [1,2], piezoelectric wafer active sensors (PWAS) [3] or entire arrays of piezoceramic modules [4] are used in many approaches. Similar applications are of sensor nodes of ultrasonic transducers [5], piezo patches [6] and, for optical measurement methods, fiber optical sensors (FOS) [7] or optical silicone-based multimode fibers [8]. Also, chip-based resistors [9] or silicon sensors [10,11] have been integrated in FRP structures. In further research contributions, foil-based flexible sensors have been developed, made of polyethylene foil [10] or polyimide foil [12,13,14,15]. Likewise, sensor layouts have been printed with conductive ink on textile fabrics, which were then processed to semi-finished products. Another possibility is the use of hybrid laminates consisting of metal and a thermoplastic melt filled with piezoceramic powder. A film of melt was spread on aluminum sheets and integrated in FRP parts [16].

While the mentioned technologies have mainly served to detect damage, there are also technologies to monitor mechanical loads. As an example, strain gauges were distributed over the structure [7,17,18]. Comparable applications did the sensing by glass fibers with an electrically conductive sizing of carbon nano tubes (CNT) [19], piezoelectric fibers made from polyvinylidene fluoride (PVDF) [20], or strain-sensitive carbon fibers [21], and CNT yarns [22]. For another application foil strain gauges were developed, which consist of piezoresistive CNT yarn [23]. Fiber Bragg gratings (FBG) (e.g., [24,25]) and phase array ultrasonic sensors [24] were used as well. Other existing technologies to monitor loads include the comparative vacuum monitoring (CVM) (Structural Monitoring Systems Ltd, Perth, Australia) or buckypaper [26].

Also, environmental loads such as temperature and humidity can be measured by piezo sensors and actuators. A possible application is their combination with transponders and radio-frequency identification application-specific integrated circuits (RFID-ASIC) [27]. For a similar application, stretchable sensor networks of pressure sensors and temperature sensors were applied to a structure [28]. Humidity can also be detected by sensors made from polyimide foil with a sensitive dielectric, which are part of a composite structure [15]. Another development is the SMARTprofile made of single mode optical fibers. It combines strain and temperature sensing in one composite coiled profile [29].

To realize process monitoring, the use of the SMARTweave method with fiber-based flat electrodes is common [30]. Alternative technologies use grids of several dielectrical sensors [31], carbon fibers or also buckypaper [26]. In another application, the use of specifically developed two-wire sensors or fringing electric field (FEF) sensors was mentioned [30]. Also, (micro-)thermocouples (e.g., [30,32]), optical fiber refractometers (OFR) [25,30,33,34], optical fiber interferometers (OFI) [30,34,35,36] or (fiber-optical) spectrometers [30,34,37,38] were integrated into structural parts. Other possible sensors are direct current resistance (DCR) sensors [38], conductive filaments [36], or micromeshes [30].

Touching on the current state of research gives an impression on the variety of technological approaches for features, which are realized by sensor integration. For composites, the monitoring of loads and damage is essential, because damage is not always identified by visual inspection. An important area of application is aircraft construction. By the entry of FRP structures into the automobile construction, this is an added topic of further focus for vehicle parts.

### 2.2. Sensor Integration: Concept

Unlike many other approaches, in the present research, the sensor technology for the integration was not specifically developed. Instead, an automotive acceleration sensor as a serial product was integrated into a structure; the sensor is currently bolted to the metal vehicle body. The advantage is given by the combination of a proven automotive sensor and the material advantages of FRP structures. This allows future functionalized automobile parts for which existing vehicle features are directly used and extended. The sensors already fulfill the requirements of the current automotive application. In addition, their sensing functionality is well known. As a benefit, the developing and validation effort is reduced concerning the sensing itself. Conversely, from a system perspective, the correct application of the currently bolted sensor is mandatory, even if the sensor is integrated into the vehicle structure. This was proven within the study covering the sensing functionality as well as the mechanical performance of integrated structures [39].

By a holistic approach, a compatible manufacturing process for structural parts with an integrated acceleration sensor was developed first. As an essential requirement, the integration process should not cause an interference of the sensor functionality. Moreover, it was determined that the sensor-integrated structures were good quality parts [39,40,41].

### 2.3. Sensor Integration: Practical

**Figure 1:**
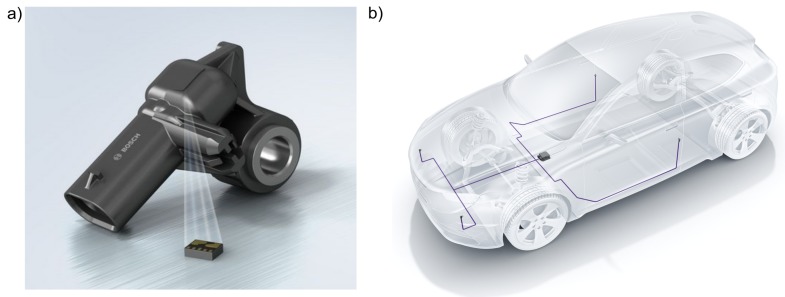
Automotive peripheral acceleration sensor: (**a**) outside housing and inside sensor module, (**b**) concept of the passive vehicle safety (schematical).

The measuring unit of the current automotive acceleration sensor is a part of the sensor module, which is inside a thermoplastic housing (Figure 1a). The housing has an electric interface for a connector and an overmoulded bolt to mount the sensor to the vehicle body. The sensor module is a micro electro mechanical system (MEMS) in the design of a land grid array (LGA). Inside the LGA is the sensor element which consists of interdependent comb structures with micromechanical electrodes on which seismic masses are hung up. Once an acceleration acts on the vehicle, a relative movement of the seismic masses occurs. The movement leads to a quantitatively measurable capacity change between the fixed and the movable part of the comb structure. The capacity change is converted to a voltage and increased. A radiometric voltage interface transfers the measured signal by the connector interface of the sensor housing to the control unit of the automobile [42,43]. The electric interface and the data protocol of the automotive acceleration sensor correspond to the peripheral sensor interface 5 (PSI5). PSI5 is an universal interface specification for a two-wire contacting. It is used for various automotive sensors [43]. Besides, the sensor can be read out by an additional fivefold wiring via the serial peripheral interface (SPI) [43].

As part of the passive vehicle safety acceleration sensors are mounted upfront and peripheral to the vehicle body for an impact detection (Figure 1b). Within a measurement range of ±120 g the sensors provide information on the direction and the level of an impact by measuring accelerations during a crash. Additional peripheral pressure sensors and upfront pressure tube sensors are applied to the vehicle. The control unit of the sensor system is located central to the vehicle. It is also equipped by acceleartion sensors.

**Figure 2:**
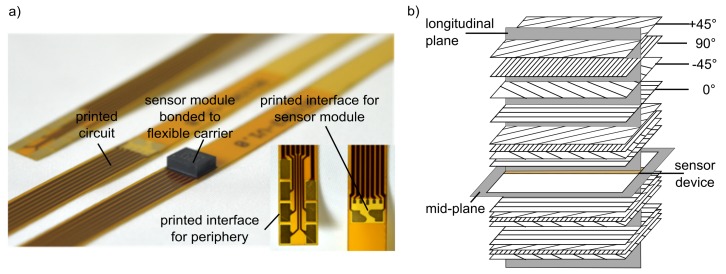
Sensor integration concept: (**a**) sensor device with flexible carrier, (**b**) laminate layup.

For the technological application, the current acceleration sensor was modified [44], in the following denoted as *sensor device*. The measuring principal of the sensor was not changed and the design of the sensor device is based on the state of the art of electronics. For the sensor module, a new packaging was developed (Figure 2a). It consists of a flexible circuit carrier of a polyimide foil substrate with a thickness of 0.1 mm. The flexible carrier has an interface for the contacting of the sensor module and an interface for the connection to the periphery, such as a standard connector. In addition, it is applied by copper tracks for the supply and data transfer of the sensor module, for both PSI5 and SPI.

The LGA design allowed an adhesive bonding of the solderable contacting pads at its bottom. Thus, the wiring of the electric contacting was eliminated and the sensor module was contacted saving space on the flexible carrier. For the adhesive mounting, isotropic conductive adhesive was used. A flipchip assembly on current pick-and-place-equipment did the assembling; an automated reel-to-reel process can later replace it.

For the target application, an integration of the sensor in FRP automotive parts, the sensor device has different advantages. Firstly, on the flex carrier, the sensor module is orientated and fixed precisely within the structure. Secondly, the flexible carrier can follow various shapes, which gives geometrical flexibility for the sensor-integrated part. Thirdly, the size of the sensor device is clearly reduced compared to the current sensor. Also, the bonding of a number of sensor modules is possible on only one flexible carrier, which additionally reduces the amount of individual wiring. By this, the necessary construction space does not significantly increase with a rising number of sensors.

The manufacturing process of structures with the integrated sensor device used resin transfer molding (RTM). RTM is one common technology of the liquid composite molding (LCM) for composite structures. Yet, the sensor device is also suitable for other LCM technologies [45]. Using a special tooling technology [44], 4 mm thick structural plates made from glass fiber fabrics were manufactured. Four layers of a quadraxial grid fabric (+45/90/−45/0) were used in a symmetrical laminate layup. The sensor device was inserted in the mid plane of the stack (Figure 2b).

### 2.4. Technology Application: Validation

Initially, it was essential to validate the technology application for the sensor’s primary function as part of the passive vehicle safety. Firstly, an evaluation of the quality and the mechanical performance of structures with an integrated sensor device was done. Secondly, the functionality of the integrated sensor was analyzed to some extent for the essential sensing functions. It was ensured that under operation conditions, no random influence on the sensor measurement was given by the surrounding FRP structure [39,46]. Thirdly, the study provided evidence that environmental conditions during operation did not have an effect on the sensing functionality longterm. Details on the research aspects are available in [39,40,41,46].

## 3. Condition Monitoring: Methods and Materials

Given the fact of an achievable technology application, the topical focus of the present research is the multiple use of the integrated automotive acceleration sensor. Next to the primary function as part of the passive vehicle safety, the sensor shall function for a condition monitoring of its surrounding structure. A cause of a conditional change is damage. It can occur during the production of a composite structure, or during operation due to loads within the structural plane or impacts. Typical modes of damage are delamination, matrix cracks, a detaching of matrix and fiber or fiber breaks [47].

### 3.1. Methodology

The method to indicate a change in structural conditions by the integrated automotive acceleration sensor is based on a reference comparison [48]. The reference system is the integrated structure in the initial condition. After a damaging event takes place, the new structural condition is the comparative system. A structural damage potentially exists if the systems show significant differences. The test concept consists of several steps. As a base, a reference signal is recorded already from the structural part in the initial state. The measured signal of the integrated sensor is a reaction to a test excitation. After the event, the signal of the sensor is recorded by the same procedure. The comparison of the signals serves to find out whether defined factors differ significantly. As an applied assumption, a change in signal behavior due to a replicated test excitation is coupled to a condition change of the surrounding structure. The assumption is because the structure and the sensor device, as an integrated part, form one integral system.

For damage detection, the signal of the automotive acceleration sensor is used. The signal reproduction is the measured acceleration over time. The typical reactions of the sensor signal to a test excitation are consecutive signal amplitudes in positive and negative physical directions, which are fading as the system levels off. For the purpose of the reference comparison, a systematic procedure for the analysis of the acceleration signal is developed.

### 3.2. Signal Analysis

The design and current use of the automotive acceleration sensor do not really focus on the targeted reference comparison. Hence, because of the measurement characteristics, the identification of differences between the compared systems might be limited by the acceleration signal given in the time domain. To avoid this, a spectral analysis of the acceleration signal is done. Since the condition of a structural part correlates to its dynamic mechanical behavior, a change in condition should manifest in a changed vibration behavior of the part.

As a starting point for the spectral analysis, the discrete sensor signal in the time domain is represented in the discrete frequency domain. A mathematical description is the discrete fourier transformation (DFT) according to Equation (1)

(1) $$a\left(t\right)=C+a_{1}sin\left(w_{1}\cdot t\right)+a_{2}sin\left(w_{2}\cdot t\right)\ldots \to X\left(f\right)=\int_{-\infty }^{\infty }x\left(t\right)\cdot e^{-j2\pi \mathrm{ft}}dt$$

Because it is arithmetically quicker, the fast fourier transformation (FFT) is used; its properties are comparable to the DFT. The FFT is done according to Equation (2)

(2) $$x_{k}=\sum_{n=0}^{n-1}X_{n}e^{-j\pi \frac{2kn}{N}}$$

N: number of interpolation points of the analyzed spectrum (extended by zero-padding)

n: number of interpolation points of the signal in the time domain

Before the FFT, a zero-padding of the time domain signal is done. The extension of the signal sequence by zeroes samples the spectrum more narrowly. Therefore, the signal transformation leads to a better representation of the spectrum and local maxima can be found more precisely. Due to the periodicity of the signal, the evaluation of the frequency spectrum takes place over one period only. After the FFT, the spectrum of the signal period lies symmetrically to the center. Thereby, for the reference comparison, a one-sided evaluation is sufficient; only the real signal components are compared. The current acceleration sensor has a low-pass filter with a cut-off frequency. Consequently, for the reference comparison, only the range within 0 Hz and the cut-off frequency is of interest.

### 3.3. Demonstrator

**Figure 3:**
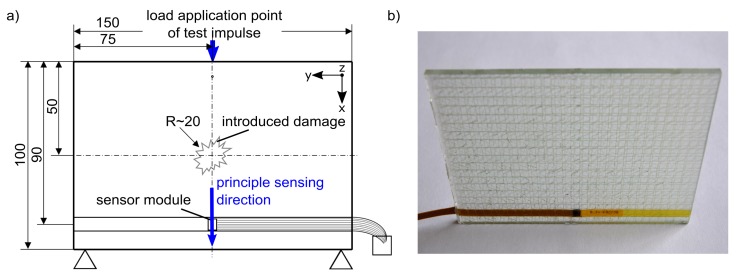
Demonstrator: (**a**) dimensions and boundary conditions of the test excitation (schematical), (**b**) test plate of experimen (not clamped in test device).

The demonstrator for the reference comparison corresponds to a lab model with a simple geometry. The demonstrator is a thin plate in which one sensor device is integrated (Figure 3). In the experiment, the test excitation is applied by an impulse of a drop weight. For this purpose, the support of the plate is vertically free at two spots along its long edge (Figure 3a). The sensor device inside the demonstrator is placed 10 mm above the supported edge. The sensor module, as part of the sensor device, is located in the middle over the width of the plate. In the middle of the opposite edge is the load application point of the impulse. The sensor device is aligned to have the principal sensing direction of the sensor module with the impulse direction. The later damage of the demonstrator is precisely introduced on the surface of the plate in its center by an impact damage.

### 3.4. Simulation

For a feasibility check of the experiment, an analysis is done on the vibration behavior of the demonstrator via the finite element method (FEM). The geometry of the numerical model is simplified and essentially corresponds to the dimensions of the demonstrator. The model is discretized by 3D solid hex elements. Orthotropic material properties are used. Focuses of the analysis are the numerical results locally at the spot at which, in the real demonstrator, the sensor module is located. The boundary conditions of the model correspond to the support and the load application point of the experiment. To avoid a complex modelling of the damage, simplistically, a hole is modeled in the numerical model. The hole has the same position and a comparable radius to the extent of the real damage. This is not appropriate with the real damaged condition. Yet, the FEM analysis primarily serves a qualitative feasibility check of the experiment and a simplistic numerical model is applicable.

#### Modal Analysis

By the numerical simulation, the eigenmodes of the demonstrator are determined by a modal analysis. The analysis follows for the undamaged model (reference system) and the hole-damaged model (comparative system).

**Figure 4:**
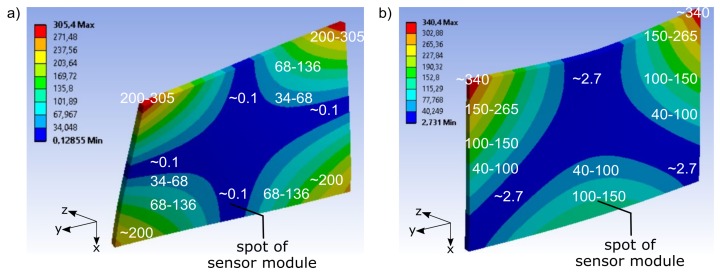
Numerical modal analysis, distribution of total deformation of undamaged demonstrator; arbitrary scaling: (**a**) first torsion mode, (**b**) first bending mode.

**Figure 5:**
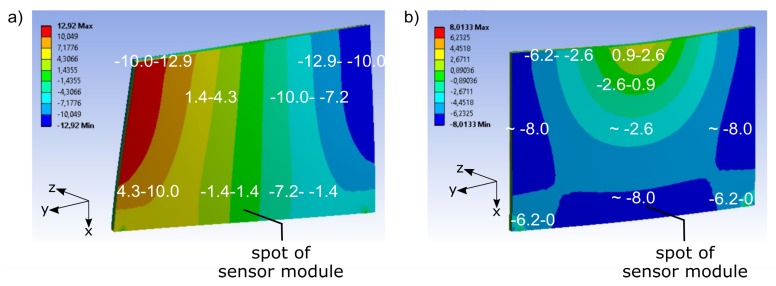
Numerical modal analysis, deformation of undamaged demonstrator in the principal sensing direction of the sensor module (x-dimension); arbitrary scaling: (**a**) first torsion mode, (**b**) first bending mode.

Within the range of 0 Hz to the cut-off frequency of the low-pass filter, the modal analysis results in two eigenmodes of the demonstrator. Firstly, the first torsion mode (Figure 4a and Figure 5a). It appears in the undamaged model at 271 Hz, and in the damaged model at 268 Hz. Secondly the first bending mode (Figure 4b and Figure 5b), in the undamaged model at 313 Hz and in the damaged model at 306 Hz. Both eigenmodes are dominant in z-dimension. In-plane (x/y-dimensions in Figure 4 and Figure 5), the effect of the eigenmodes is relatively small. The sensing acts in this plane, and the principal sensing direction of the sensor corresponds to the x-dimension. Locally, equivalent to the spot at which in the real demonstrator the sensor module is located, the eigenmodes manifest themselves differently. Figure 4 shows the relative distribution of the total deformation over the demonstrator for each eigenmode. The torsion mode (Figure 4a) has a rather small effect at the spot of the sensor module, compared to its effects at other spots of the demonstrator. The bending mode (Figure 4b) affects the spot of the sensor module more significantly with respect to the other spots.

It is interesting how the eigenmodes affect the principal sensing direction of the sensor module. Thus, the deformation in the x-dimension is evaluated. Figure 5 shows the maximum deformation in the x-dimension for each eigenmode. It equally shows that, at the local sensor module, the torsion mode (Figure 5a) manifests itself at comparatively low levels related to other spots of the demonstrator. The bending mode has a noticeable effect, compared to the other spots (Figure 5b). The effects of the deformation are qualitatively similar in the undamaged and damaged model for both eigenmodes. Similar to the undamaged condition, in the damaged condition, the effect at the spot of the sensor module is more significant by the bending mode.

The modal analysis enabled an assessment of the characteristics of the eigenmodes at the given frequencies within the range of 0 Hz to the cut-off frequency of the low-pass filter of the integrated sensor. This provides a general understanding of how the demonstrator will react once the excitation is applied by the test impulse. In the following step, experiments will be done on real test plates.

### 3.5. Experiments

For the demonstrator test plates were cut out of the glass fiber reinforced polymer (GFRP) structural plates which were manufactured by the RTM. Each test plate has one integrated sensor device (Figure 3). The part of the flex carrier with the periphery interface is fed out laterally. GFRP has the advantage of transparency, thus the extent of the later damage can be identified especially on the inside of the structure. This allows an optical assessment to confirm a damage, which is indicated by the signal analysis.

The measurement series are repeatedly done on five test plates. The use of several test plates ensures that the interpretation of the signal analysis is tolerant to structural imperfections, which might occur during the production of composite structures. Due to this, scattering of part quality is common to some extent and might also appear in the target application. For the reference comparison, the test plates will later be impact-damaged in the center of the plate (Figure 3a).

#### 3.5.1. Test Set-Up

The test excitation is applied by an impulse of a guided drop weight on a specially developed test device. The essential requirement is a reproducible, defined excitation of the demonstrator. The demonstrator must preferably be able to oscillate freely as a reaction to the excitation. For the vertical excitation, the test plate is positioned on a knife-edge support and partially point supported. The support is decoupled from the test impulse device. Influences by the natural oscillation of the support is eliminated by measures of vibration isolation. The drop weight contacts the test plate only as it strikes the load application point. The part of the sensor device, which is fed out of the test plate, is connected to a PSI5 Simulyzer USB box (SesKion, Leinfelden–Echterdingen, Germany). It allows to control and to read out the integrated sensor module.

The experiments are done with a drop height of 15 mm, which generates an impulse of 0.8 kg m/s. By the impulse, the demonstrator is accelerated with 100 m/s${}^{2}$ by the impingement. The acceleration is adequate for the test; it is much higher than the signal noise of the sensor. The test setup produces an excitation frequency on the demonstrator of around 260 Hz.

#### 3.5.2. Measurements

**Figure 6:**
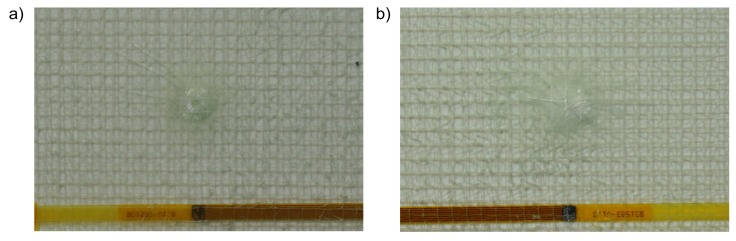
Test plate of experiment after impact-damaging (**a**) upper face, (**b**) lower face.

First, the test excitation is applied to the test plate in undamaged condition. The signal of the sensor device is continuously read out and recorded. The measurement is pursued by the impact-damaging of the demonstrator (Figure 6). The impact is applied with an energy of 10 J. The measurements are then repeated for the damaged test plates by the same procedure.

## 4. Results

Initially an assessment of the experimental measurements is done by the acceleration signal in the time domain. As a first result it is obvious, that the deviation of the acceleration signal over time does not exceed the typical range of signal noise of the current acceleration sensor; this confirms sufficient repeatability. For the reference comparison, the systematic signal analysis is carried out for the measured signals. Contrasting the experimental results with the numerical results of the FEM simulation (Section 3.4), a feasibility check is followed.

### Analysis Results

**Figure 7:**
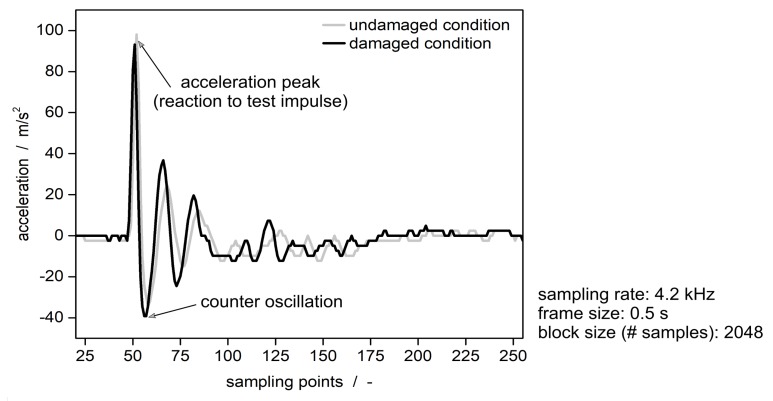
Acceleration signal in the time domain after zero-padding, in undamaged and damaged condition.

The signal of the sensor device in the time domain nominally returns the applied acceleration of around 100 m/s${}^{2}$ as a reaction to the test excitation (Figure 7). It has the typical signal characteristics of the acceleration signal of the sensor due to an external excitation. Comparing the demonstrator in undamaged and damaged condition there is no difference between the time signals. The magnitude of the acceleration (ordinate in Figure 7) is identical within the measuring accuracy for both conditions. Also, the cycle time of the first full wave (abscissa in Figure 7) is comparable for both conditions. The other signal wave form shows the typical fading oscillation.

**Figure 8:**
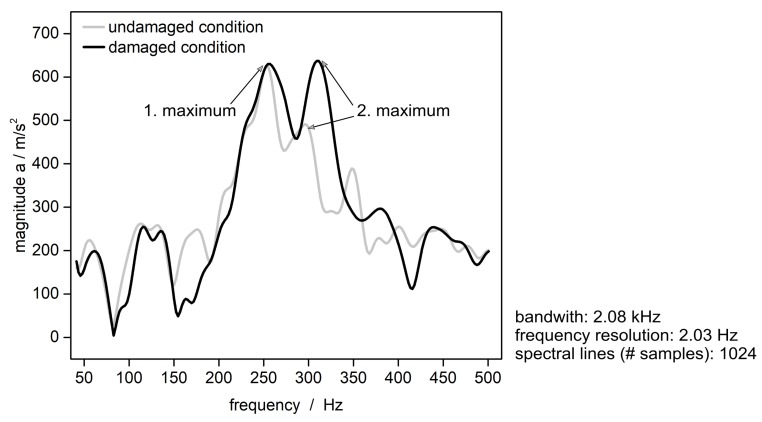
Envelope curves of the spectral components of the acceleration signal, in undamaged and damaged condition.

The signal analysis provides other findings. In the frequency domain, the comparison of the acceleration signals shows a clear effect by the damage. The envelope curves of the spectral components of the acceleration signal show the difference respectively before and after the damage (Figure 8).

The envelope curves of the signal of both the undamaged and the damaged demonstrator, have two clear maxima of increased frequency components of the spectrum. Table 1 gives the position (frequency) and the magnitude of the first maximum, Table 2 of the second maximum. Also the nominal delta related to the undamaged condition (reference system) is given.

It appears that the damage of the demonstrator causes no significant shift in position of the maximum frequency components for the first maximum (Table 1, column 2) as well as the second maximum (Table 2, column 2). For both maxima, the position varies between the undamaged and damaged condition within the standard deviation. The maximum frequency components which appear in the signal of the experiment were similarly displayed by the numerical modal analysis (Section 3.4). The numerical model has its first torsion mode at 271 Hz in undamaged condition, and at 268 Hz in hole-damaged condition. The frequencies are comparable to the position of the first maximum in Table 1. The first bending mode of the numerical calculation appears at 313 Hz in an undamaged condition, and at 306 Hz with a hole. The frequencies are comparable to the position of the second maximum in Table 2.

The magnitudes of the maximum frequency components are interesting. The magnitude of the first maximum (torsion mode) shows no significant difference between the undamaged and the damaged condition; quantitatively, the deviation lies within the standard deviation (Table 1, column 3). Since the test excitation was applied by a frequency of around 260 Hz, it can be assumed that the spectral components at this frequency are a direct representation of the test excitation. Because it was applied consistently durint the testing of the demonstrator in both conditions, there is no difference between the corresponding magnitude of the frequency components of the sensor signals. Already, the FEM simulation had showed that the torsion mode has only marginal effects locally at the spot of the sensor module. To put it briefly, a change in condition manifests only slightly in the first torsion mode of the demonstrator. A remarkable difference shows the magnitude of the second maximum (bending mode). After the damage, the magnitude changes +17% (Table 2, column 3). The increase is distinctive; it greatly exceeds the standard deviation. The effect is validated by the results of the FEM analysis. The simulation showed that the bending mode, which appears around this frequency, manifests noticable locally at the spot of the sensor module. To sum up, the change in condition of the structure manifests clearly in the first bending mode of the demonstrator.

As a conclusion, the experiment proves the applied assumption. It demonstrates that a condition change of the surrounding structure is indicated by a different signal behavior of the integrated sensor device. As the signal in the time domain represents the externally applied acceleration without noticeable changes between both conditions, undamaged and damaged, the representation of the signal in the frequency domain shows a clear difference in characteristics. Because the damage causes a reduced stiffness of the structure, an excessive displacement appears. This associates with increased maximum frequency components at the first bending mode of the demonstrator. The latter appears in the spectrum (second maximum) of the acceleration signal of the sensor device. The fact that specifically the second maximum is affected by the condition change is validated by the FEM modal analysis. The FEM showed, that the bending mode has a noticeable effect at the spot of the sensor modul. The position of the second maximum in the experiment is equivalent to the first bending mode of the simulation; the minor discrepancy between the frequency values can be justified by the simplistic modelling of the FEM. A complementary numerical acceleration excitation analysis can serve for a better understanding, especially to compare the displacements of the damaged and the undamaged demonstrator. Likewise, to go ahead with the obtained findings, further studies are useful on various demonstrators, in particular, of geometrical complexity.

## 5. Discussion

As an integrated sensor device, the automotive acceleration sensor is an integral part of the structure and forms an entire system with the automotive part. The present research showed that the acceleration signal can indicate a change in condition of the structure. The functionality is provided if the sensor is integrated at a spot of the structure where, as a minimum, one eigenmode of the structure locally manifests itself. In the present research, the relevant modes affected the demonstrator by a minor amount in the principal sensing direction of the integrated sensor. Yet, the sensor signal represented changes of the modes by sufficient accuracy to detect the damage. An optimized alignment of the sensor device within the structure might raise the resolution. As a premise, the vibration behavior of the sensor-integrated structure must be known within the sensing range initially. To further refine the condition monitoring for the target application, several distributed sensors could be integrated into one automotive part. The design of the sensor device makes this easily possible as it allows an application of several sensor modules on only one flexible carrier. Due to its flexibility, the sensor device is also suitable for an integration in structures of geometrical complexity. As a benefit, the secondary feature can be realized without developing a special sensor technology, and without attaching additional sensors to the vehicle body. It has to be mentioned that an exhaustive unrestricted functionality for the primary function of the passive vehicle safety is subjected to a comprehensive validation of the integrated sensor. In the frame of the study, the essential functions of the integrated sensor were examined to some extent but not for any load acquired during vehicle operation.

## 6. Conclusions

This research contribution introduces a new perspective for the application of the current automotive acceleration sensor. As an integrated device, the sensor can initiate a new use case which leads the way for future automotive FRP lightweight design. The application does not only provide a solution to use a current safety system for future FRP vehicles, it also serves for a secondary safety feature which is essential for FRP structures. During operation, damage to FRP vehicle parts might occur by stone chipping or smaller collisions but might not clearly be identifiable; thus, condition monitoring is mandatory. Prospective, as a third feature for future automobiles the reference comparison could serve for a qualitiy check of FRP vehicle parts right after or during their production [49]. Damage of a composite structure can already occur during the manufacturing process caused by an insufficient impregnation, inclusions or cavities; those can be indicated by the integrated sensor.

Prior to this, it is conceivable to determine the degree of curing of the FRP structure while it is being produced. During the LCM, the successive hardening of the resin correlates with an increasing stiffness of the FRP structure. Thereby, the structure changes its vibration behavior during the curing process. This can be identified by the reference comparison of the sensor signals at certain process states. Regardless of the reference comparison, the present research also showed that the sensor signal analysis is rather precise to detect specific signal characteristics. Hence, the integrated sensor device might also serve as an indicator at various other manufacturing process steps. As an example, during resin injection, the signal analysis of the accelearation signal might state the specific event when the liquid resin arrives at the sensor module on the inside of the mold. This helps to track the flow of the resin during the injection process, determining a sufficient mold filling. Pre-studies on this research topic have been done, but more detailed studies are necessary.

All things considered, the present research indicates an opportunity to realize multiple usage of the integrated automotive acceleration sensor as a serial product for FRP automotive structures. In fact, the features realized by the integrated automotive sensor might also be useful for FRP structures beyond the automotive sector, whereat the development of new sensors is not intended [50].

## Figures and Tables

**Table 1:** Frequency and magnitude of **1. maximum** of increased frequency components for the demonstrator in undamaged and damaged condition; median (*std*) for frequency, magnitude and delta.

Condition of Demonstrator	Frequency [Hz]	Magnitude [m/s${}^{2}$]
undamaged	257 (*2.5*)	680 (*19.7*)
damaged	245 (*10.6*)	646 (*30.6*)
related delta	−0.04 (*0.03*)	−0.03 (*0.03*)

**Table 2:** Frequency and magnitude of **2. maximum** of increased frequency components for the demonstrator in undamaged and damaged condition; median (*std*) for frequency, magnitude and delta.

Condition of Demonstrator	Frequency [Hz]	Magnitude [m/s${}^{2}$]
undamaged	305 (*5.5*)	535 (*10.5*)
damaged	308 (*9.5*)	627 (*25.9*)
related delta	0 (*0.02*)	0.17 (*0.03*)

## Abbreviations

The following abbreviations are used in this manuscript:

DFT	Discrete fourier transformation
FEM	Finite element method
FFT	Fast fourier transformation
FRP	Fiber reinforced polymer
GFRP	Glas fiber reinforced polymer
LGA	Land grid array
MEMS	Micro electro mechanical system
PSI5	Peripheral sensor interface 5
RTM	Resin transfer molding
SHM	Structural health monitoring
SPI	Serial peripheral interface
